# Pebble and Rock Band: Heuristic Resolution of Repeats and Scaffolding in the Velvet Short-Read *de Novo* Assembler

**DOI:** 10.1371/journal.pone.0008407

**Published:** 2009-12-22

**Authors:** Daniel R. Zerbino, Gayle K. McEwen, Elliott H. Margulies, Ewan Birney

**Affiliations:** 1 European Bioinformatics Institute, Wellcome Trust Genome Campus, Hinxton, Cambridge, United Kingdom; 2 Genome Technology Branch, National Human Genome Research Institute, National Institutes of Health, Bethesda, Maryland, United States of America; University of Maryland, United States of America

## Abstract

**Background:**

Despite the short length of their reads, micro-read sequencing technologies have shown their usefulness for *de novo* sequencing. However, especially in eukaryotic genomes, complex repeat patterns are an obstacle to large assemblies.

**Principal Findings:**

We present a novel heuristic algorithm, Pebble, which uses paired-end read information to resolve repeats and scaffold contigs to produce large-scale assemblies. In simulations, we can achieve weighted median scaffold lengths (N50) of above 1 Mbp in Bacteria and above 100 kbp in more complex organisms. Using real datasets we obtained a 96 kbp N50 *in Pseudomonas syringae* and a unique 147 kbp scaffold of a ferret BAC clone. We also present an efficient algorithm called Rock Band for the resolution of repeats in the case of mixed length assemblies, where different sequencing platforms are combined to obtain a cost-effective assembly.

**Conclusions:**

These algorithms extend the utility of short read only assemblies into large complex genomes. They have been implemented and made available within the open-source Velvet short-read *de novo* assembler.

## Introduction

Next-generation sequencing (NGS) technologies, thanks to their high throughput and cost effectiveness, present many experimental opportunities for molecular biologists. These tools are gaining wide popularity, for example in SNP detection [1] structural variant detection [2] and DNA assays [3]. However, all these methods rely on the existence of a finished reference genome onto which the data is mapped. The use of this short-read data to reconstruct, or assemble, *de novo* genomes has progressed much more slowly.

The NGS platforms currently on the market can be divided into two categories. Firstly, 454 sequencers [4]produce 400 to 500 base pair (bp) fragments, or reads. They have been used in some *de novo* studies [5]. The Illumina [6] and SOLiD [7] platforms produce much shorter reads: 50 to 70 bp and 35 to 50 bp, respectively. These micro-reads are much more difficult to assemble because their short lengths, and therefore short overlaps, prevent from distinguishing ambiguous repeat copies in a genome [8].

Despite this difficulty, assembly software has been developed to solve this problem, such as EULER-SR [9], SSAKE [10], VCAKE [11], SHARCGS [12] ALLPATHS [13], EDENA [14], ABySS [15] and our own program, Velvet [16]. These tools have shown that micro-reads can already be used to obtain draft assemblies of bacterial genomes. However, assemblies of eukaryotic genomes remain fragmented due to the complex repeats in these genomes.

To overcome this obstacle, paired-end reads have been widely considered as a promising solution. Sequencing both ends of a DNA fragment of known length produces not only two sequences but also their relative placement information, which can be exploited to constrain the space of possible assemblies. This approach has been studied in projects such as ARACHNE [17] and BAMBUS [18]. In all of these examples paired-end information was used to order and orient, or scaffold, contigs, and test the validity of these contig assemblies.

EULER-DB [19] presented the idea of extracting the sequence between contigs, even though it belonged to collapsed repeated regions. Read-pairs were tested separately to check if they could constrain the scaffolding problem by defining a unique path between two contigs. ALLPATHS [13] extended this idea by bundling all the read-pairs connecting two contigs to reduce calculations.

The SHORTY algorithm [20] additionally used sets of mate-pairs which were all anchored on one end to a unique word of length *k*, or *k*-mer. This allowed the algorithm to obtain localisation information at the scale of the insert length variance.

In our previous paper [16] we presented a simple scaffolding algorithm, Breadcrumb, which was inspired by the initial SHORTY algorithm, but used long contigs instead of *k*-mers to anchor groups of mate-pairs. Breadcrumb could resolve simpler repeats but was quickly limited in the case of mammalian genomes.

In a subsequent paper, Hossain *et al.* [21] presented improvements on the SHORTY algorithm, based on the use of either long reads or pre-computed contigs to bundle mate-pairs together. Moreover, their method now uses *a priori* knowledge of the insert length distribution to estimate the gaps between contigs.

We describe here a new read-pair resolution algorithm, Pebble, which exploits the knowledge of insert lengths to resolve more complex situations. This use of insert lengths not only allows the algorithm to localise mate-pairs much more efficiently using only short unique contigs, but is also instrumental in resolving the sequence of complex repeat copies.

Another approach to resolving repeats is through mixing long and short reads, to improve assemblies by benefiting from the respective advantages of the different technologies. We present a simple method, named Rock Band, to exploit sparse long read datasets within a short-read assembly to resolve repeats and extend contigs.

We tested our solutions on simulated datasets then extended to experimental data from *Pseudomonas syringae* and ferret. We obtained scaffold N50s above 100 kbp with few missassemblies, and compared our method to a leading alternative de Bruijn assembler, EULER-USR [22].

## Results

The Pebble and Rock Band algorithms were designed to function within the Velvet assembler, and as such are based on the de Bruijn graph structure presented in detail in [23] and [16]. In both algorithms, a number of assembly contigs, or graph nodes, are identified as being unique. Both algorithms then try to find the correct path which connect consecutive contigs.

### Identifying Unique Nodes

To resolve repeats with confidence it is necessary to determine which contigs in the assembly are unique. Various methods based on topology have been used [23], [24] but in its current implementation, Velvet only relies on contig coverage values, using a statistic derived from the A-statistic [25]. The high density of read start positions offered by next-generation sequencing data makes it easier to consider a contig coverage measurement as a sum of independent measurements, thus introducing the contig length as an added parameter (cf. Methods).

### Pebble: Using Paired-End Information

Pebble tries to connect the unique nodes identified previously, but using the paired-end information. For each unique node, chosen in an arbitrary order, it iteratively estimates distances from that node, extends it to the next unique node, then merges the distance information provided by both nodes.

#### Building a primary scaffold

Before resolving repeats, it is necessary to organise read-pair information in a condensed and convenient structure. For any two nodes in the graph, Velvet enumerates the reads or mate-pairs which connect them. Using the maximum likelihood estimator (MLE) described in [26], it estimates the distance between them. The complete set of estimated inter-node distances is called the *primary scaffold*. Because each contig is represented by two nodes, one for each strand, orientation is retained.

This approach makes a number of simplifying assumptions. Firstly, it supposes that each insert library has a normal length distribution. Secondly, it only accounts for read-pairs which successfully connect two given nodes, thus biasing the insert length distribution by interval censoring. To be exhaustive, the MLE would have to consider the likelihood of the read-pairs which fail to connect the two nodes. However, this formalization would be much more expensive to calculate, because of the quadratic number of operations, as the information of each read-pair would have to be integrated in many MLE calculations simultaneously.

#### Construction of a secondary scaffold

Before trying to extend a unique node *A*, it is necessary to establish which other nodes are in its vicinity. Pebble starts by extracting the primary scaffold information relative to *A*. However, this set of distances is generally insufficient to extend it. If the insert length is longer than *A*'s length, then there is no primary information on its immediate neighbourhood. For this reason Pebble computes a set of local distances, or *secondary scaffold*, relative to *A*.

The SHORTY [21] and Breadcrumb [16] algorithms were respectively based on the concept of projecting the image of a unique *k*-mer or a unique node onto its neighbourhood, using paired-end reads. In Pebble, the approach is slightly different: for a given unique node, we enumerate the neighbouring unique nodes which project onto it, then use those to estimate distances around it.

**Figure 1:**
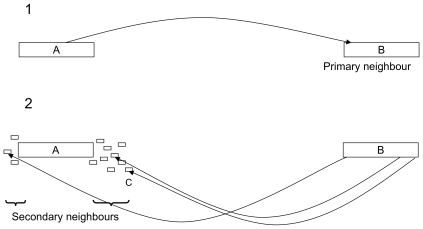
Construction of the secondary scaffold. From a unique node A, Pebble starts by incorporating all the distances relative to this node found in the primary scaffold. For every unique node B which is connected to A, Pebble then follows the primary connections associated to B, thus flagging secondary neighbours of A. Assuming that all the nodes are laid out on a line, we can estimate that the distance from A to C is equal to the distance from A to B, minus that from B to A.

Figure 1 describes the process used to construct the secondary scaffold. We call primary neighbours the unique nodes which share a connection with A in the primary scaffold. Pebble extracts all the primary connections of these nodes, and flags the nodes to which they lead, which we call *secondary neighbours*. Using the known primary distance estimates, it is possible, by a simple subtraction, to derive an estimate of the distance between *A* and the secondary neighbours.

The secondary scaffold takes into account orientation by using algebraic distances. Contigs which are upstream of the reference point are assigned negative distances, whereas contigs downstream are assigned positive ones. This notation allows Pebble to subtract distances without any constraint.

However, derived distance estimates have to be handled with caution, because secondary neighbours are not necessarily unique. It is possible that the distance estimate from a unique contig to a repeated one is actually based on the distances from the unique node to separate copies of the repeat. This means that distances in the secondary scaffold tend to be fuzzy.

#### Heuristic search through the local scaffold

Once Pebble has determined a set of distance estimates from node *A*, it attempts to find a plausible path through its secondary neighbours connecting it to the nearest unique node. The strategy employed is a heuristic depth-first search (cf. Text S1). Pebble advances through the graph, from one secondary neighbour to the next, over the existing arcs.

At each iteration, it searches through the outgoing arcs, and chooses the direct neighbour which is putatively closest to the starting point. To avoid loops, priority is systematically given to nodes which have not yet been visited, or have been visited the fewest amount of times.

If a path is found to the next unique node, then the sequence associated to the path and the sequence of the destination node are appended to the starting node. All the read information from the destination is transposed onto the starting node. The destination node can then be deleted.

Reads belonging to the path are left in place. By default the nodes along the path are considered to be potential repeats. This is why Pebble does not track the position reads within resolved repeats.

### Rock Band: Using Long Read Information

The advantage of the de Bruijn graph over the more traditional overlap graph is to allow the mixture of read-lengths. If long reads are available, they can be easily used to connect the nodes of the graph after error correction, using a simple procedure called Rock Band. The main idea is that if all the long reads which go out of one unique node go consistently to another unique node and vice-versa, then both nodes can be confidently merged.

**Figure 2:**
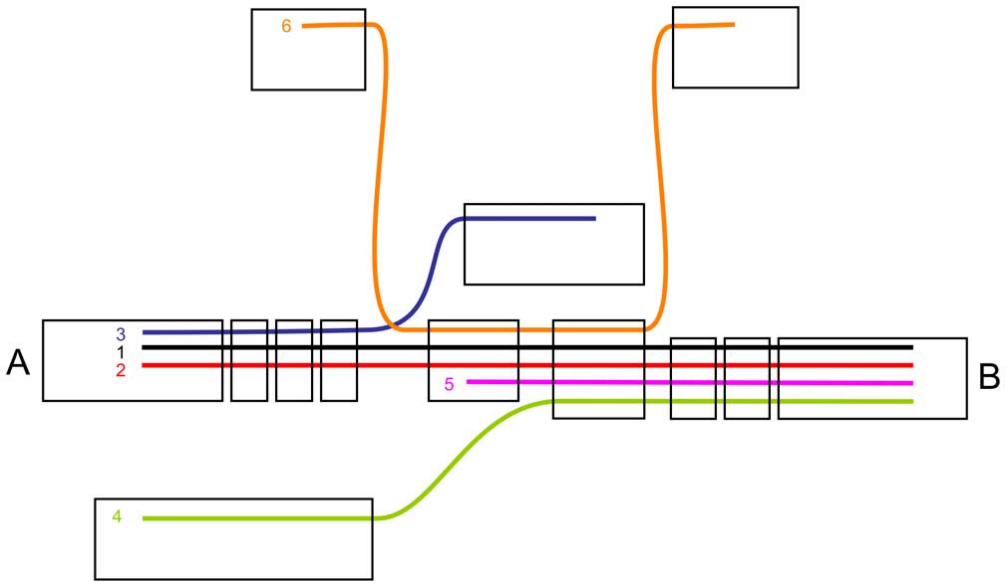
Resolution of repeats through the Rock Band assembly. In this simplified diagram, contigs are represented as boxes, and long reads as thick curved lines. Instead of performing a pair-wise comparison of reads, the algorithm only examines the reads going out of unique node A. Two of the reads (1 and 2) go to node B. Node 3 is disregarded because it is not confirmed by another read. The algorithm then examines the reads going into node B. They all come from node A, except read 4, which is disregarded because unconfirmed, and read 5 which is not in contradiction with the assembly of contigs A and B. Finally, read 6, despite its overlap with the other reads, is disregarded throughout the analysis, as it goes through neither nodes A nor B.

Examining every unique node in an arbitrary order, Rock Band iteratively tries to extend that node to the next unique node. It enumerates the long reads which go out of that node, and follows them to the next unique node, as described in Figure 2. If all the long reads go to the same destination, then Rock Band merges the two unique nodes, using the long reads' sequences to fill the gap between the two contigs.

At the next iteration, some of the reads used previously to find a path may come to an end. However, merging the nodes involves also merging the sets of reads, possibly incorporating new reads which allows the process to continue.

### Simulations

We tested our software on simulated datasets generated from genomic regions from 4 different species, namely *E. coli*, *S. cerevisiae*, *C. elegans* and *H. sapiens*. To factor out genome length, we chose a 5 Mb region from each of these genomes, except for *E. coli* where the whole 4.8 Mb genome was used.

Randomly placed reads were generated according to three different scenarios. In the first case, perfect reads were generated from the reference sequence. In the second, single substitution base-pair errors were inserted at a rate of 1%. Finally, we simulated a diploid sequencing project. We generated an alternate version of the reference where artificial SNPs were introduced randomly at a rate of 1 per 500 bp. One half of the reads was generated from the original reference, the other from the modified reference. All reads contained random errors as above.

#### Paired-end assemblies

**Figure 3:**
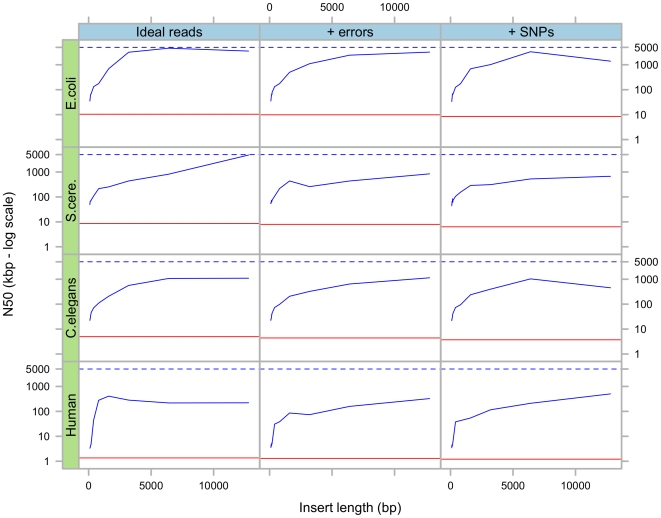
Results of simulations using various insert lengths. Final scaffold N50 as a function of insert length, in four different species and three different simulations scenarios. The horizontal red lines represent the initial N50 after error removal and before repeat resolution. The dashed blue lines represent the highest possible N50, namely the length of the sequence being sampled.

In the first simulation, we tested various insert lengths, from 100 bp to 12kb in short read format (35 bp) alone. The results in Figure 3 show that as the insert length increases, the N50 also increases, until it stabilises, and eventually decreases. The initial rise is simply due to the increased insert lengths, thus increasing the probability for nearby unique regions to be connected by mate-pairs. The final degradation is linked to the higher variance of the insert lengths, which can cause Pebble to detect possible misassemblies and interrupt its progression (cf. Methods).

Pebble can integrate multiple read pair libraries, but the length of the scaffolds produced is governed mainly by the size of the longest library as long as the variance in the insert length is reasonable, in practice around 10% of the insert length. Larger variance in insert sizes degrades the N50.

#### Mixed-length assemblies

**Figure 4:**
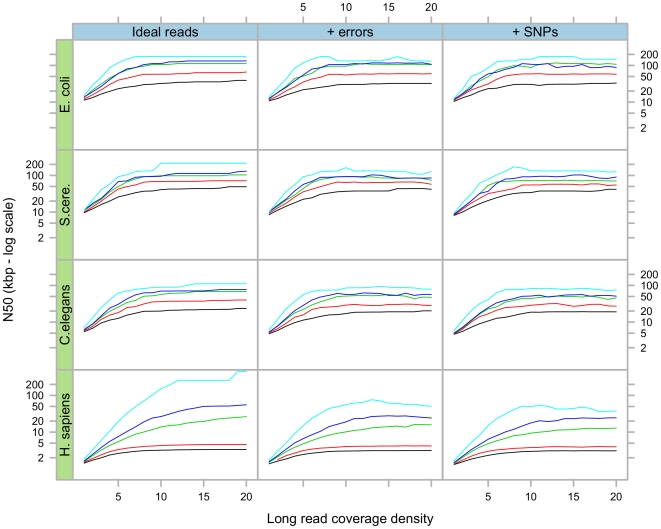
Results of simulations using various long/short read mixtures. Final contig N50 as a function of long read concentration, in four different species, and three different simulation scenarios. The length of the long reads is represented by the colour of the curves: 100 (black) 200 (red) 400 (green) 500 (blue) and 1000 bp (light blue).

In the second experiment, we tested various mixtures of long and short reads. The results, shown in Figure 4, are qualitatively as one would expect. The more long reads are available, the more repeats are covered, the more contigs are merged together. Similarly, as long reads get longer, then the number of repeats which can potentially be bridged goes up, so the resulting contigs are longer.

However there is a fixed ceiling for each read length, due to the repeat structure of long repeats in genomes. The level and behaviour of this ceiling varies according to the genome and the length of the long reads. For *E. coli*, *S. cerevisiae* and *C.elegans* this ceiling is reached at a similar coverage depth, and it increases regularly with the length of the reads. However, in human there is a sharp distinction between 200 bp reads on one hand and 400 and 500 bp reads on the other. The former are shorter than Alu sequences and produce an N50 below 5 Kbp, whereas the latter allow the construction of much longer contigs. Because of the density of repeats in the human genomes, a higher coverage of long reads is required to reach the ceiling.

In the case of reads with errors, at very high coverage depths the N50 diminishes. This would be due to the accumulation of errors which become more difficult to distinguish from genuine sequence, and then prevent the Rock Band algorithm from resolving repeats.

**Figure 5:**
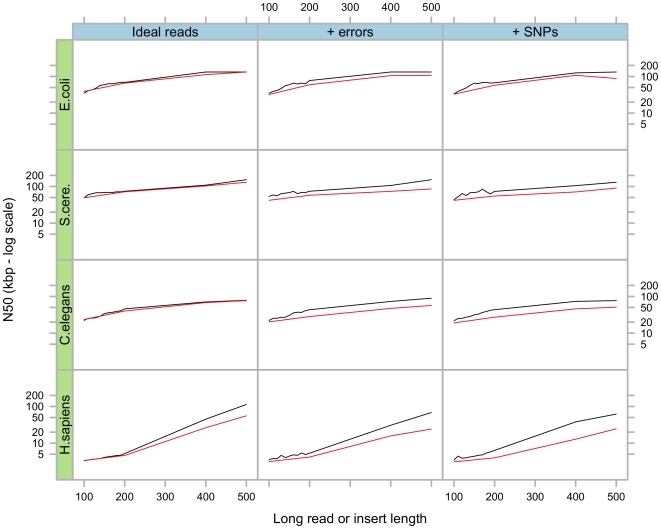
Comparison of the Rock Band and Pebble methods. The red and black curves represent the final scaffold N50 after the execution of the Rock Band or Pebble algorithms respectively, as a function of long read or insert length, in four different species and three different simulations scenarios, as described in figures 3 and 4.

Higher N50 contig lengths were obtained in this experiment (∼1 Mbp in bacteria, ∼100 Kbp in human) than in the previous simulations with mixed length reads. This difference is due to the fact that inserts can be much longer than long reads. Pebble is able to effectively exploit these very long insert lengths through the construction of the secondary scaffold. However, for comparable lengths, high density paired-end reads consistently produce slightly longer scaffolds than long reads, as shown on Figure 5. This is due to the fact that in these simulations, the physical density of paired-end reads is much higher than that of long-reads (in this case 20x). This result argues in favour of dense read-pairs to resolve repeats over long reads. In our experience, longer reads do not significantly improve assemblies, as the insert length is the principal factor which determines the quality of the assembly.

### Sequencing *Pseudomonas syringae*

Pebble was tested on actual Illumina data, generated from *Pseudomonas syringae* [27]. 6073272 36 bp reads were collected with an average insert length of 400 bp. After running Velvet, 274 contigs longer than 100 bp where obtained, with an N50 supercontig length of 104 kbp and a maximum supercontig length of 314 kbp. The run took in total 5 min 38 s and required 1.2 GB of RAM.

Only 15 short contigs did not align to the reference, representing a total of 26 kbp. 10 of those contigs represented the copies of a common repeat, which did not align perfectly to the reference genome. The other contigs only aligned partially to the reference, and presented novel sequence, possibly due to minor contamination. For example, one of them aligned to a *Xanthomas campestris* insertion sequence. None of them presented a concatenation of distant *Pseudomonas* sequences.

If scaffolding is turned off then the contig N50 goes down to 24 kbp. However, many of the buffers are very short. If the contigs are only broken up at buffers which are estimated to be strictly longer than 1 bp, then the N50 of the “near-contigs” becomes 48 kbp

These results were compared to those obtained with EULER-USR [23]. EULER was found to be the leading paired-end micro-read assembler [15]. The ALLPATHS assembler [13] could not be used because it is designed to function with two different insert lengths, unlike Velvet which can handle one or several libraries indifferently.

The details of this comparison are in Table 1. Whereas Euler does produce longer contigs, Velvet's near-contigs are comparable to Euler's contigs. Moreover, Velvet's scaffolds are significantly longer.

**Table 1:** Comparison of Velvet and EULER assemblies.

Assembly	N50 length (kbp)	Maximum length (kbp)	Contig or scaffold count	Coverage (%)
EULER contigs	40	215	598	103.0
Velvet contigs	24	134	595	97.9
Velvet near-contigs	48	157	430	97.9
EULER scaffolds	51	215	620	107.8
Velvet scaffolds	96	314	274	98.4

Comparison of Velvet and Euler assemblies. The contig or scaffold count corresponds to the number of contigs or scaffolds longer than 100 bp. Near-contigs are defined as scaffolds which are broken up only if the distance between two contigs is estimated to be strictly greater than 1 bp.

### Sequencing Ferret BACs

Finally, Pebble was tested on Illumina data from a ferret BAC. 690,494 36+36 bp read pairs were generated with a 270 bp insert length. This assembly produced a single contig 147.362 bp long, which aligned with a 0.02% global error rate. These results show that microreads can be used to obtain contigs of significant length even on complex genomes.

## Discussion

Pebble is a new de Bruijn graph algorithm which can use read pairs to extend genomic assembly contigs using the dense paired-end information produced by next-generation sequencing platforms. We tested this method on simulated as well as experimental datasets. The length weighted median contig length (N50) in simulations is within that reached in traditional “draft” assemblies, and for all genomes is greater than the median gene length. Although not the focus of this paper, a light coverage of longer insert lengths (such as fosmid end pairs) will be able to easily super-scaffold these scaffold-contigs into even longer collinear components. Thus using only short reads, useful assemblies can be generated for complex organisms.

The RockBand algorithm fills a similar role using single long reads added to the genome. Long reads in this context might be from 454 data, or traditional Sanger reads, with not enough coverage for assembly by other means. In many cases there are already partial long read datasets which can be leveraged now with the addition of short reads. For reads as long as short-read paired-end inserts, Rock Band and Pebble produces comparable N50s. However, Pebble is also able to resolve repeats using longer insert lengths. This study confirms the intuition of many genomic researchers that the generation of longer length inserts (3 Kbp-10 Kbp) will be very valuable for resolving genomic features.

The Pebble algorithm requires tracking of each short read through the error correction processes of tip clipping and TourBus described in our previous paper. This tracking information produces a large memory requirement, with 120 Mbp assemblies needing 50 to 100 GB of physical memory. This means that currently the main limitation for using Velvet on larger genomes are engineering (rather than algorithmic) issues. The ABySS de Bruijn graph system works on a distributed memory system and can handle larger genomes [15]. However, resolving the complex repeat structures at such a scale and producing long contigs is still a difficult task. The next focus will be to improve the engineering to provide a robust, large-scale de Bruijn assembler.

In this paper we have explored these algorithms by simulation and with two real datasets. These algorithms have been available in partial form in the Velvet package for around one year, and a variety of other groups have already reported good results with these methods, such as [27]. There is a growing community of researchers who are using Velvet regularly, and these algorithms extend the utility of this tool.

## Materials and Methods

### Pebble Implementation

#### Removing false connections in the primary scaffold

After establishing for each unique node a list of its primary neighbours, Pebble removes connections between unique nodes which are not supported by enough evidence. To start with, it discards distance estimates which were derived from less than a given cutoff (by default 4) of mate-pairs. Assuming that the distance estimate is correct, it then estimates the expected number of mate-pairs which should connect the two contigs (cf. below). If the observed number is below 10% of that expected values, then the distance estimate is deleted.

#### Estimating the expected number of paired-end connections between two contigs

For each pair of contigs connected by read pairs, and for each insert library, we call *A* the length of the longer node, *B* the length of the shorter one, *D* the estimated distance between the two, and *ρ* the density of paired-end reads on the longer node. The paired-end reads are characterised by the mean *μ* and the standard deviation *σ* of the insert length distribution.

We first define a few variables:
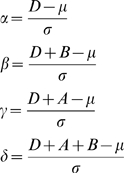



We finally obtain an estimate of the expected number *X* of paired-end connections between the two contigs (cf. Text S1), using the probability density *ϕ* and the standard error function *erf* associated to the normal distribution:
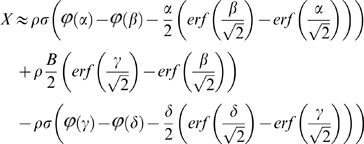



#### Scaffold merging

As two unique nodes are being merged into one, it is vital to also merge the corresponding scaffolding information, to keep the process going. This operation is directly based on the original construction of the local scaffold, since the formula allows us to incrementally add information, both from primary and derived distances.

Admittedly this use of the formula ignores the fact that the derived distances around one node are not necessarily independent from those around a neighbouring node. Nonetheless, this bias is neglected for the sake of computational speed.

#### Interrupted searches

It is not always possible to extend a node to another unique contig. This can be due to coverage gaps, or to anomalies detected by Pebble as described below. However, the search for a path between two contigs is asymmetric, depending on which of the two the local scaffold was built around.

Whenever Pebble estimates that two contigs, using read-pair information, should be neighbours, but fails to find a path from both ends, it finally scaffolds the two together. They are merged as if a path were found, but separated by a buffer sequence of undetermined nucleotides (marked as N's). The length of this buffer is equal to the distance estimate between the nodes.

#### Avoiding misassemblies

Because Pebble is a greedy algorithm, special care must be taken to avoid creating misassemblies. These can be detected through inconsistencies in contig distances. When extending a unique contig, Pebble checks that it terminates on the nearest unique contig in the local scaffold, and did not step directly to a farther one. Such inconsistencies can be caused by the variance in the distance estimator, especially when using mate-pair libraries with high insert length variance. However, for the sake of precaution, Pebble stops whenever it meets such an occurrence.

#### Avoiding infinite loops

Another pitfall of this heuristic depth-first search is looping within highly repetitive regions of the graph before eventually finding the correct path out. In its current implementation, Pebble does not try to resolve these regions, but simply jumps over them.

To prevent looping, Pebble stops whenever it visits the same node twice, without having visited a new node in between.

### Rock Band Implementation

#### Identifying unique nodes

We denote by *X_i_* the number of read starts on *k-*mer *i* of a given contig and assume that the distribution of *X_i_* is a Poisson distribution of mean and variance both equal to the expected density *ρ*. In practice, this expected density is determined empirically, after running the first stages of Velvet. The distribution of the average contig coverage depths is used to set the expected coverage either visually with a histogram or with a formula such as the length-weighted median.

We assume that the multiplicity within each contig's *k*-mers is constant, because of the properties of the de Bruijn graph. In other words, a node is either unique or repeated *r* times, but it should not be a mixture of unique and repeated *k*-mers.

According to the Central Limit Theorem, the mean of all the *X_i_* inside a node of length *n*, follows a distribution which converges towards a normal distribution of mean *ρ* and standard deviation 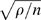. To decide whether a node is unique or not, we calculate the log-odds ratio of the node being unique over that of it being twice in the genome. Given an observed mean density of 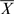, we thus obtain the function:
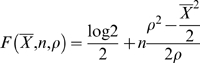



To ensure specificity, we impose a uniqueness cutoff of *F*≥5. For practical reasons, the short read coverage is used as a proxy for the number of short reads, on the assumption that the short reads have a near constant length.

#### Dealing with errors and variation

To reduce calculations, long reads are clipped at their ends so that all their tips are mapped onto unique nodes. This clipping can potentially completely erase long reads which are apparently included in repeated regions. Nonetheless, this is quite rare, as in our experience, even within eukaryotic repeats short unique regions can be found.

To reduce noise, a path between two unique nodes is neglected if it is not validated by two reads or more. When confronted with discrepancies between long reads, Rock Band interrupts its progression, and flags the corresponding node as non-unique.

### Paired-End Simulations

Random 35 bp paired-end short reads were generated at a constant coverage of 50x. The simulated insert lengths were either: 100, 200, 300, 400, 500, 600, 700, 800, 1600, 3200, 6400, or 12800 bp long. The actual insert lengths were randomly varied around the expected value with a standard deviation of 10% of that length.

### Mixed-Length Simulations

In this set of simulations, random 35 bp short reads were generated at a constant coverage of 50x. Random long reads were generated at varying coverage values, namely all integer values from 1 to 20x. The long reads were either 100, 200, 400, 500 or 1000 bp long.

### *P. syringae* Sequencing

The 36 bp reads from *Pseudomonas syringae* are available in the Short Read Archive under accession number ERA000095 and came from lane 7 of run 20708_20H04AAXX_R1 on machine ID49. They were assembled using *k-*mer length 21 bp and the following parameters, “-cov_cutoff 7 -exp_cov 13 –ins_length 400”.

To set these parameters, we ran several assemblies without any options other than a varying k-mer length, and chose the one which produced the highest N50 length. From that preliminary assembly, 13x was the mode of the contig coverage distribution, and 7x was chosen as just above half the previous value. Finally, the insert length was determined by the fragment length selection performed by the authors of the experiment [27].

The contigs were aligned to the reference using exonerate [28] with options “-m ner –bestn 1”. Contigs which were found to have an alignment onto the reference at least 50 bp shorter than their actual length were then examined using BLASTZ [29].

### Ferret Sequencing

The ferret Illumina dataset is available in the Short Read Archive under the accession number SRA009025. The 36 bp reads correspond to sample 149, run on lanes 3 and 4 of flowcell 30GTEAAXX (2 December 2008). The BAC corresponds to clone CH237-509L18, accession number AC170700. Velvet was run with a hash length of 31 bp and the following parameters, “-cov_cutoff 20 -exp_cov 40 -ins_length 270”.

### Availability

Velvet is implemented in C, and has been tested on various Linux, MacOS and Sparc/Solaris systems. It is freely available under GPL 2 license at www.ebi.ac.uk/~zerbino/velvet. The package includes a manual which describes the parameter setting.

## Supporting Information

Text S1 This additional text provides more details on the methods described in the main manuscript.
(0.23 MB DOC)
