# Supplementary material for: Pebble and Rock Band: Heuristic Resolution of Repeats and Scaffolding in the Velvet Short-Read de Novo Assembler
Source: PLoS One. 2009 Dec 22;4(12):e8407. doi: 10.1371/journal.pone.0008407 (PMC2793427; doi:10.1371/journal.pone.0008407)
Supplement: Text S1 — This additional text provides more details on the methods described in the main manuscript. (0.23 MB DOC) [file pone.0008407.s001.doc]

# Supplemental material

## Pebble pseudo-code

**Integrate distances into secondary scaffold**(Unique node N):

For each primary connection C attached to N:

A := destination of C

Add distance information to local scaffold:

d*(X,A) ← d(X,A)

If (A is unique):

For each primary connection C* attached to A:

B := destination of C*

Add distance information to local scaffold:

d*(X,B) ← d(X,A) – d(A,b)

For each primary connection C attached to Ñ:

… analogous to previous loop …

**Find a path to the next unique node**(Starting node N):

Current node := N

While(TRUE):

Destination := ∅

For each arc A of current node:

Candidate := destination of A

If (Destination := ∅

OR Count(Candidate) < Count(Destination)

OR (Count(Candidate) == Count(Destination) AND distance(Candidate) < distance(Destination)):

Destination := Candidate

If (loop detected)

Return FAIL

If (missassembly detected)

Return FAIL

Append sequence of Destination to Node

Current := Destination

If (Destination is unique)

**Integrate distances into local scaffold**(Destination)

Return SUCCESS

**Pebble**:

**Identify unique nodes** ()

**Compute primary scaffold** ()

Do:

For each unique node N:

**Clear local scaffold**()

**Integrate distances into local scaffold** (N)

Do:

**Find a path to the next unique node**(N)

Until (fails)

Until (converges)

##

## Estimating the expected number of mate-pairs which should connect two nodes

We wish to estimate to how many mate-pairs would connect two nodes A and B, assuming that the distance between the two is known.

For this calculation we will only we using the length of A and B (which will also be written A and B for clarity), the distance D separating them, and the number of reads on each of them. Renaming the nodes if necessary, we assume that A is longer than B. To obtain the expected number of connecting mate-pairs, we will calculate the probability for a read r on A to connect to B, then multiply it by the number of reads on A.

Assuming that the distribution of reads is uniform over the length of A, *P* can be expressed as a sum of probabilities each depending on the coordinate p_r_ of the start of r on node A:

P can then be transformed into a sum of probabilities on the insert length of r, *l(r)*:

Assuming that the probability distribution of *l*(*r*) is a known normal function of mean *μ* and variance *σ*^2^, its probability distribution function (pdf) is noted $j_{m,s}$:

where S(a,b,x) returns 1 if a ≤ x ≤ b and 0 otherwise. Summing the terms under the integration sign, we then find an easily computable approximation:

where F(A,B,D,x) is the continuous function, linear by parts, which returns (cf. fig. 1):

- 0 if x ≤ D
- (x-D) if D ≤ x ≤ D+B
- B if D+B ≤ x ≤ D+A
- (A+B+D-x) if D+A ≤ x ≤ D+A+B
- 0 if D+A+B ≤ x


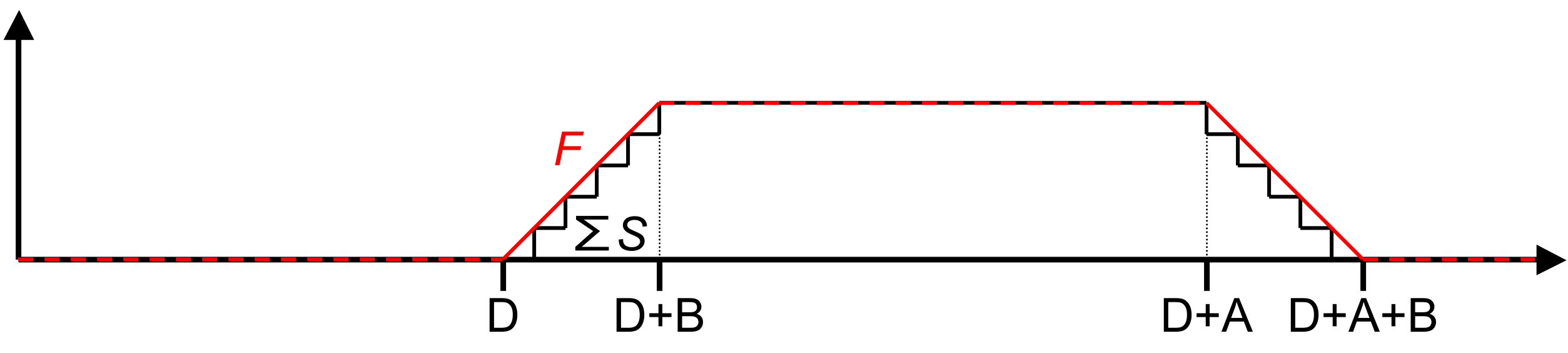


Figure 1: Schematic diagram of *S* and *F* functions

Changing variables with $u=\frac{x-m}{s}$ the expression becomes, using $j$ as pdf associated to *N*(0,1):

Defining new parameters:

we obtain:

Using the integrative properties of ϕ, we finally obtain:

where *erf* is the standard error function associated to the normal distribution. We thus calculate P and multiply it by the number of reads on A to obtain an approximate expected number X of mate-pair connections between the two nodes, using ρ as the density of reads on A:

The advantage of this formula is that a result can be quickly calculated from just seven numerical parameters. Unfortunately, the error of the approximation could not be bounded.

Instead, tests were run on experimental Illumina data from the sequencing of Pseudomonas syringae (Farrer et al., 2009). The expected insert length length was 400bp, so connections between unique nodes were judged correct or incorrect depending on whether the distance between the contigs on the reference was smaller or greater than 1000bp. Correct connections which were flagged as correct but with fewer than 4 supporting read pairs, were discarded as they would be in the pipeline. This filter was not applied to the incorrect connections because very few would have passed this second selection. The results of this test are displayed in figure 2. The estimated value was very close to the observed count in the case of the correct connections.

After removing connections for which the number of read pairs was below 10% of the expected value, only 0.38% of the retained connections were erroneous, and only 1 correct connection was incorrectly filtered out. In this example all but one of these false positive hits would not have been selected because the number of connecting read pairs was below the default cutoff (= 4). The addition of this filter was observed to significantly reduce the number of misassemblies created by the Pebble algorithm.


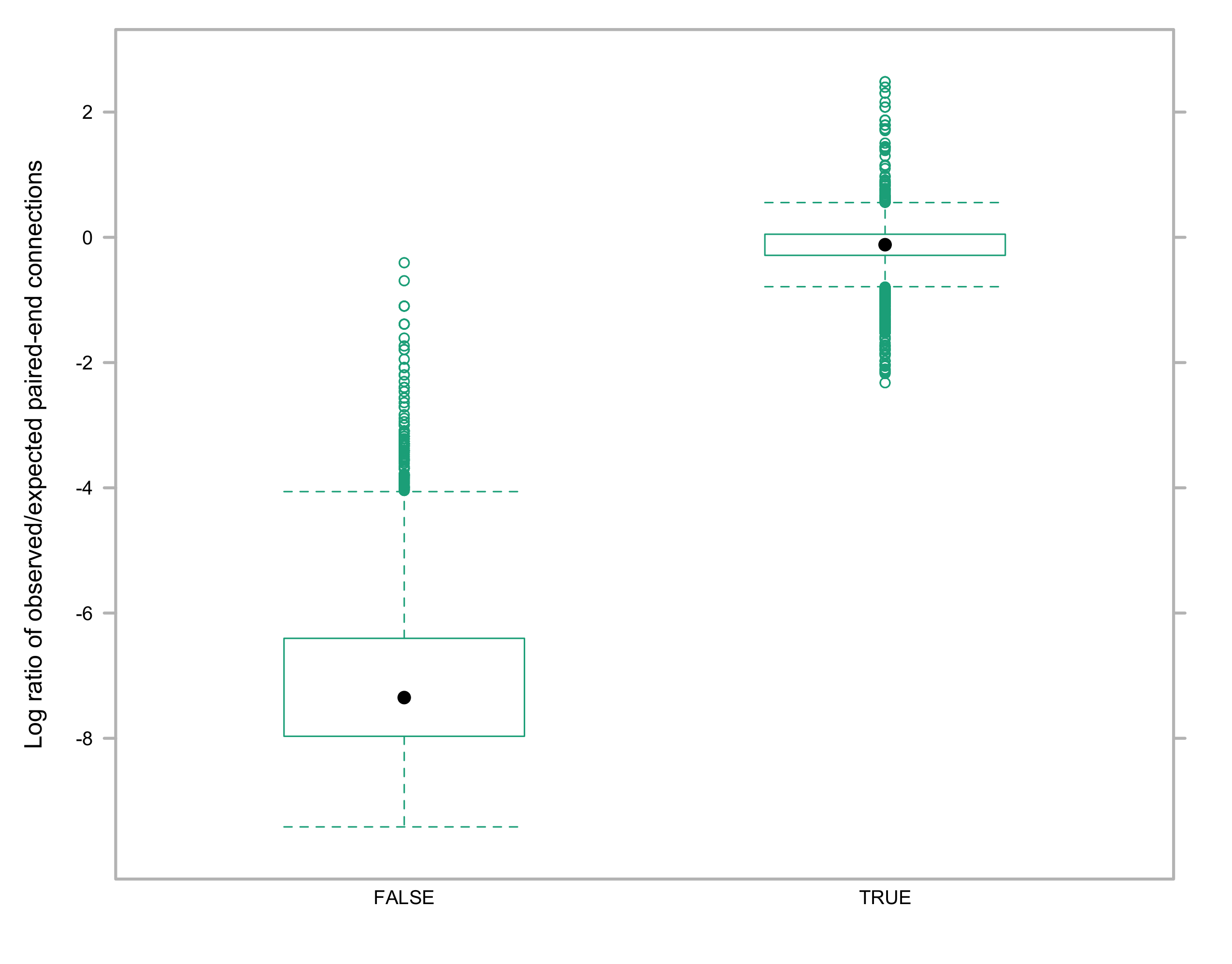


Figure 2: Test of the paired-end multiplicity estimator on experimental data.

Unique contigs from the *P. syringae* assembly were queried for paired-end read connections. If the contigs in a connection were mapped less than 1kb apart, it was flagged as correct, otherwise it was flagged as incorrect. This boxplot represents the log-ratio of the number of observed divided by the number of expected connecting read pairs.
